# On the scope of scientific hypotheses

**DOI:** 10.1098/rsos.230607

**Published:** 2023-08-30

**Authors:** William Hedley Thompson, Simon Skau

**Affiliations:** ^1^ Department of Applied Information Technology, University of Gothenburg, Gothenburg, Sweden; ^2^ Institute of Neuroscience and Physiology, Sahlgrenska Academy, University of Gothenburg, Gothenburg, Sweden; ^3^ Department of Pedagogical, Curricular and Professional Studies, Faculty of Education, University of Gothenburg, Gothenburg, Sweden; ^4^ Department of Clinical Neuroscience, Karolinska Institutet, Stockholm, Sweden

**Keywords:** hypotheses, metascience, psychology, philosophy of science

## Abstract

Hypotheses are frequently the starting point when undertaking the empirical portion of the scientific process. They state something that the scientific process will attempt to evaluate, corroborate, verify or falsify. Their purpose is to guide the types of data we collect, analyses we conduct, and inferences we would like to make. Over the last decade, metascience has advocated for hypotheses being in preregistrations or registered reports, but how to formulate these hypotheses has received less attention. Here, we argue that hypotheses can vary in specificity along at least three independent dimensions: the relationship, the variables, and the pipeline. Together, these dimensions form the scope of the hypothesis. We demonstrate how narrowing the scope of a hypothesis in any of these three ways reduces the hypothesis space and that this reduction is a type of novelty. Finally, we discuss how this formulation of hypotheses can guide researchers to formulate the appropriate scope for their hypotheses and should aim for neither too broad nor too narrow a scope. This framework can guide hypothesis-makers when formulating their hypotheses by helping clarify what is being tested, chaining results to previous known findings, and demarcating what is explicitly tested in the hypothesis.

## 1. Introduction

Hypotheses are an important part of the scientific process. However, surprisingly little attention is given to hypothesis-making compared to other skills in the scientist's skillset within current discussions aimed at improving scientific practice. Perhaps this lack of emphasis is because the formulation of the hypothesis is often considered less relevant, as it is ultimately the scientific process that will eventually decide the veracity of the hypothesis. However, there are more hypotheses than scientific studies as selection occurs at various stages: from funder selection and researcher's interests. So which hypotheses are worthwhile to pursue? Which hypotheses are the most effective or pragmatic for extending or enhancing our collective knowledge? We consider the answer to these questions by discussing how broad or narrow a hypothesis can or should be (i.e. its scope).

We begin by considering that the two statements below are both hypotheses and vary in scope:

H_1_: For every 1 mg decrease of *x*, *y* will increase by, on average, 2.5 points.
H_2_: Changes in *x*_1_ or *x*_2_ correlate with *y* levels in some way.

Clearly, the specificity of the two hypotheses is very different. H_1_ states a precise relationship between two variables (*x* and *y*), while H_2_ specifies a vaguer relationship and does not specify which variables will show the relationship. However, they are both still hypotheses about how *x* and *y* relate to each other. This claim of various degrees of the broadness of hypotheses is, in and of itself, not novel. In *Epistemetrics,* Rescher [1], while drawing upon the physicist Duhem's work, develops what he calls *Duhem's Law.* This law considers a trade-off between certainty or precision in statements about physics when evaluating them. Duhem's Law states that narrower hypotheses, such as H_1_ above, are more precise but less likely to be evaluated as true than broader ones, such as H_2_ above. Similarly, Popper, when discussing theories, describes the reverse relationship between content and probability of a theory being true, i.e. with increased content, there is a decrease in probability and vice versa [2]. Here we will argue that it is important that both H_1_ and H_2_ are still valid scientific hypotheses, and their appropriateness depends on certain scientific questions.

The question of hypothesis scope is relevant since there are multiple recent prescriptions to improve science, ranging from topics about preregistrations [3], registered reports [4], open science [5], standardization [6], generalizability [7], multiverse analyses [8], dataset reuse [9] and general questionable research practices [10]. Within each of these issues, there are arguments to demarcate between confirmatory and exploratory research or normative prescriptions about how science *should* be done (e.g. science is ‘bad’ or ‘worse’ if code/data are not open). Despite all these discussions and improvements, much can still be done to improve hypothesis-making. A recent evaluation of preregistered studies in psychology found that over half excluded the preregistered hypotheses [11]. Further, evaluations of hypotheses in ecology showed that most hypotheses are not explicitly stated [12,13]. Other research has shown that obfuscated hypotheses are more prevalent in retracted research [14]. There have been recommendations for simpler hypotheses in psychology to avoid misinterpretations and misspecifications [15]. Finally, several evaluations of preregistration practices have found that a significant proportion of articles do not abide by their stated hypothesis or add additional hypotheses [11,16–18]. In sum, while multiple efforts exist to improve scientific practice, our hypothesis-making could improve.

One of our intentions is to provide hypothesis-makers with tools to assist them when making hypotheses. We consider this useful and timely as, with preregistrations becoming more frequent, the hypothesis-making process is now open and explicit*.* However, preregistrations are difficult to write [19], and preregistered articles can change or omit hypotheses [11] or they are vague and certain degrees of freedom hard to control for [16–18]. One suggestion has been to do less confirmatory research [7,20]. While we agree that all research does not need to be confirmatory, we also believe that not all preregistrations of confirmatory work must test narrow hypotheses. We think there is a possible point of confusion that the specificity in preregistrations, where researcher degrees of freedom should be stated, necessitates the requirement that the hypothesis be narrow. Our belief that this confusion is occurring is supported by the study Akker *et al*. [11] where they found that 18% of published psychology studies changed their preregistered hypothesis (e.g. its direction), and 60% of studies selectively reported hypotheses in some way. It is along these lines that we feel the framework below can be useful to help formulate appropriate hypotheses to mitigate these identified issues.

We consider this article to be a discussion of the researcher's different choices when formulating hypotheses and to help link hypotheses over time. Here we aim to deconstruct what aspects there are in the hypothesis about their specificity. Throughout this article, we intend to be neutral to many different philosophies of science relating to the scientific method (i.e. how one determines the veracity of a hypothesis). Our idea of neutrality here is that whether a researcher adheres to falsification, verification, pragmatism, or some other philosophy of science, then this framework can be used when formulating hypotheses.^1^

The framework this article advocates for is that there are (at least) three dimensions that hypotheses vary along regarding their narrowness and broadness: the selection of relationships, variables, and pipelines. We believe this discussion is fruitful for the current debate regarding normative practices as some positions make, sometimes implicit, commitments about which set of hypotheses the scientific community ought to consider good or permissible. We proceed by outlining a working definition of ‘scientific hypothesis' and then discuss how it relates to theory. Then, we justify how hypotheses can vary along the three dimensions. Using this framework, we then discuss the scopes in relation to appropriate hypothesis-making and an argument about what constitutes a scientifically novel hypothesis. We end the article with practical advice for researchers who wish to use this framework.

## 2. The scientific hypothesis

In this section, we will describe a functional and descriptive role regarding how scientists use hypotheses. Jeong & Kwon [21] investigated and summarized the different uses the concept of ‘hypothesis’ had in philosophical and scientific texts. They identified five meanings: assumption, tentative explanation, tentative cause, tentative law, and prediction. Jeong & Kwon [21] further found that researchers in science and philosophy used all the different definitions of hypotheses, although there was some variance in frequency between fields. Here we see, descriptively*,* that the way researchers use the word ‘hypothesis’ is diverse and has a wide range in specificity and function. However, whichever meaning a hypothesis has, it *aims to be true, adequate, accurate or useful* in some way.

Not all hypotheses are ‘scientific hypotheses'. For example, consider the detective trying to solve a crime and hypothesizing about the perpetrator. Such a hypothesis still aims to be true and is a tentative explanation but differs from the scientific hypothesis. The difference is that the researcher, unlike the detective, evaluates the hypothesis with the scientific method and submits the work for evaluation by the scientific community. Thus a *scientific hypothesis* entails a *commitment to evaluate the statement with the scientific process*.^2^ Additionally, other types of hypotheses can exist. As discussed in more detail below, scientific theories generate not only scientific hypotheses but also contain auxiliary hypotheses. The latter refers to additional assumptions considered to be true and not explicitly evaluated.^3^

Next, the scientific hypothesis is generally made *antecedent* to the evaluation. This does not necessitate that the event (e.g. in archaeology) or the data collection (e.g. with open data reuse) must be collected before the hypothesis is made, but that the evaluation of the hypothesis cannot happen before its formulation. This claim state does deny the utility of exploratory hypothesis testing of *post* *hoc* hypotheses (see [25]). However, previous results and exploration can generate new hypotheses (e.g. via abduction [22,26–28], which is the process of creating hypotheses from evidence), which is an important part of science [29–32], but crucially, while these hypotheses are important and can be the conclusion of exploratory work, they have yet to be evaluated (by whichever method of choice). Hence, they still conform to the antecedency requirement. A further way to justify the antecedency is seen in the practice of formulating a *post hoc* hypothesis, and considering it to have been evaluated is seen as a questionable research practice (known as ‘hypotheses after results are known’ or HARKing [33]).^4^

While there is a varying range of specificity, is the hypothesis a critical part of all scientific work, or is it reserved for some subset of investigations? There are different opinions regarding this. Glass and Hall, for example, argue that the term only refers to falsifiable research, and model-based research uses verification [36]. However, this opinion does not appear to be the consensus. Osimo and Rumiati argue that any model based on or using data is never wholly free from hypotheses, as hypotheses can, even implicitly, infiltrate the data collection [37]. For our definition, we will consider hypotheses that can be involved in different forms of scientific evaluation (i.e. not just falsification), but we do not exclude the possibility of hypothesis-free scientific work.

Finally, there is a debate about whether theories or hypotheses should be linguistic or formal [38–40]. Neither side in this debate argues that verbal or formal hypotheses are not possible, but instead, they discuss normative practices. Thus, for our definition, both linguistic and formal hypotheses are considered viable.

Considering the above discussion, let us summarize the scientific process and the scientific hypothesis: a hypothesis guides what type of data are sampled and what analysis will be done. With the new observations, evidence is analysed or quantified in some way (often using inferential statistics) to judge the hypothesis's truth value, utility, credibility, or likelihood. The following working definition captures the above:

*Scientific hypothesis*: an implicit or explicit statement that can be verbal or formal. The hypothesis makes a statement about some natural phenomena (via an assumption, explanation, cause, law or prediction). The scientific hypothesis is made antecedent to performing a scientific process where there is a commitment to evaluate it.

For simplicity, we will only use the term ‘hypothesis’ for ‘scientific hypothesis' to refer to the above definition for the rest of the article except when it is necessary to distinguish between other types of hypotheses. Finally, this definition could further be restrained in multiple ways (e.g. only explicit hypotheses are allowed, or assumptions are never hypotheses). However, if the definition is more (or less) restrictive, it has little implication for the argument below.

## 3. The hypothesis, theory and auxiliary assumptions

While we have a definition of the scientific hypothesis, we have yet to link it with how it relates to scientific theory, where there is frequently some interconnection (i.e. a hypothesis tests a scientific theory). Generally, for this paper, we believe our argument applies regardless of how scientific theory is defined. Further, some research lacks theory, sometimes called convenience or atheoretical studies [41]. Here a hypothesis can be made without a wider theory—and our framework fits here too. However, since many consider hypotheses to be defined or deducible from scientific theory, there is an important connection between the two. Therefore, we will briefly clarify how hypotheses relate to common formulations of scientific theory.

A scientific theory is generally a set of axioms or statements about some objects, properties and their relations relating to some phenomena. Hypotheses can often be deduced from the theory. Additionally, a theory has boundary conditions. The boundary conditions specify the domain of the theory stating under what conditions it applies (e.g. all things with a central neural system, humans, women, university teachers) [42]. Boundary conditions of a theory will consequently limit all hypotheses deduced from the theory. For example, with a boundary condition ‘applies to all humans’, then the subsequent hypotheses deduced from the theory are limited to being about humans. While this limitation of the hypothesis by the theory's boundary condition exists, all the considerations about a hypothesis scope detailed below still apply *within* the boundary conditions. Finally, it is also possible (depending on the definition of scientific theory) for a hypothesis to test the same theory under different boundary conditions.^5^

The final consideration relating scientific theory to scientific hypotheses is auxiliary hypotheses. These hypotheses are theories or assumptions that are considered true simultaneously with the theory. Most philosophies of science from Popper's background knowledge [24], Kuhn's paradigms during normal science [44], and Laktos' protective belt [45] all have their own versions of this auxiliary or background information that is required for the hypothesis to test the theory. For example, Meelh [46] auxiliary theories/assumptions are needed to go from theoretical terms to empirical terms (e.g. neural activity can be inferred from blood oxygenation in fMRI research or reaction time to an indicator of cognition) *and* auxiliary theories about instruments (e.g. the experimental apparatus works as intended) and more (see also Other approaches to categorizing hypotheses below). As noted in the previous section, there is a difference between these auxiliary hypotheses, regardless of their definition, and the scientific hypothesis defined above. Recall that our definition of the scientific hypothesis included a *commitment* to evaluate it. There are no such commitments with auxiliary hypotheses, but rather they are assumed to be correct to test the theory adequately. This distinction proves to be important as auxiliary hypotheses are still part of testing a theory but are separate from the hypothesis to be evaluated (discussed in more detail below).

## 4. The scope of hypotheses

In the scientific hypothesis section, we defined the hypothesis and discussed how it relates back to the theory. In this section, we want to defend two claims about hypotheses:

(A1) Hypotheses can have different *scopes*. Some hypotheses are narrower in their formulation, and some are broader.
(A2) The scope of hypotheses can vary along three dimensions relating to *relationship selection*, *variable selection*, and *pipeline selection*.

**Figure 1.**
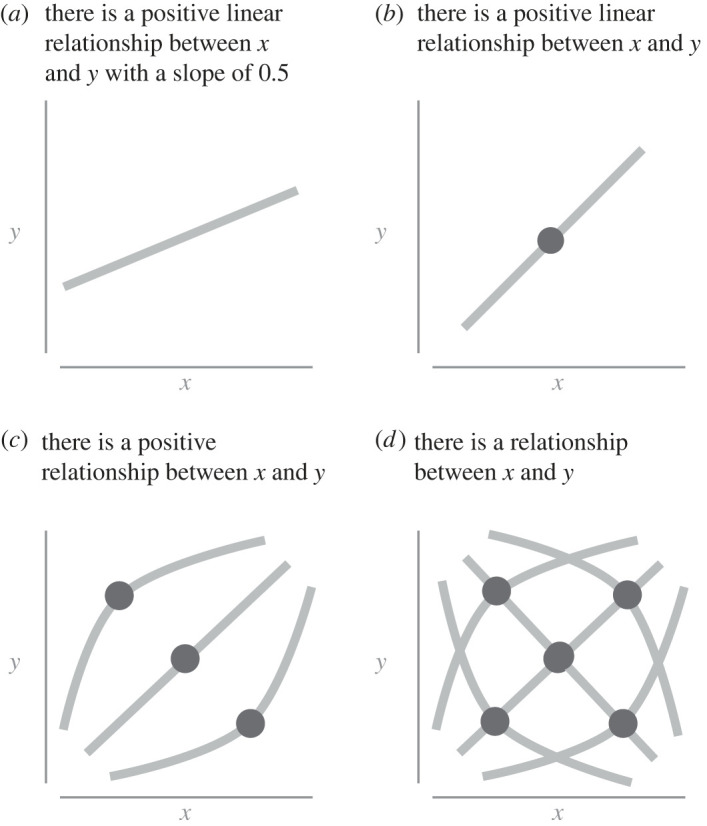
Examples of narrow and broad hypotheses between *x* and *y*. Circles indicate a set of possible relationships with varying slopes that can pivot or bend.

A1 may seem obvious, but it is important to establish what is meant by *narrower* and *broader* scope. When a hypothesis is very narrow, it is specific. For example, it might be specific about the type of relationship between some variables. In figure 1, we make four different statements regarding the relationship between *x* and *y*. The narrowest hypothesis here states ‘there is a positive linear relationship with a magnitude of 0.5 between *x* and *y*’ (figure 1*a*), and the broadest hypothesis states ‘there is a relationship between *x* and *y*’ (figure 1*d*). Note that many other hypotheses are possible that are not included in this example (such as there being no relationship).

We see that the narrowest of these hypotheses claims a type of relationship (linear), a direction of the relationship (positive) and a magnitude of the relationship (0.5). As the hypothesis becomes broader, the specific magnitude disappears (figure 1*b*), the relationship has additional options than just being linear (figure 1*c*), and finally, the direction of the relationship disappears. Crucially, all the examples in figure 1 can meet the above definition of scientific hypotheses. They are all statements that can be evaluated with the same scientific method. There is a difference between these statements, though—*they differ in the scope of the hypothesis*. Here we have justified A1.

Within this framework, when we discuss whether a hypothesis is *narrower* or *broader* in scope, this is a relation between two hypotheses where one is a subset of the other. This means that if H_1_ is narrower than H_2_, and if H_1_ is true, then H_2_ is also true. This can be seen in figure 1*a–d*. Suppose figure 1*a*, the narrowest of all the hypotheses, is true. In that case, all the other broader statements are also true (i.e. a linear correlation of 0.5 necessarily entails that there is also a positive linear correlation, a linear correlation, and some relationship). While this property may appear trivial, it entails that it is only possible to directly compare the hypothesis scope between two hypotheses (i.e. their broadness or narrowness) where one is the subset of the other.^6^

### 4.1. Sets, disjunctions and conjunctions of elements

The above restraint defines the scope as relations between sets. This property helps formalize the framework of this article. Below, when we discuss the different dimensions that can impact the scope, these become represented as a set. Each set contains elements. Each element is a permissible situation that allows the hypothesis to be accepted. We denote elements as lower case with italics (e.g. *e*_1_, *e*_2_, *e*_3_) and sets as bold upper case (e.g. **S**). Each of the three different dimensions discussed below will be formalized as sets, while the total number of elements specifies their scope.

Let us reconsider the above restraint about comparing hypotheses as narrower or broader. This can be formally shown if:

*e*_1_, *e*_2_, *e*_3_ are elements of **S_1_**; and
*e*_1_ and *e*_2_ are elements of **S_2_**,

then **S_2_** is narrower than **S_1_**.

Each element represents specific propositions that, if corroborated, would support the hypothesis. Returning to figure 1*a*,*b*, the following statements apply to both:

‘There is a positive linear relationship between *x* and *y* with a slope of 0.5’.

Whereas the following two apply to figure 1*b* but not figure 1*a*:

‘There is a positive linear relationship between *x* and *y* with a slope of 0.4’ (figure 1*b*).
‘There is a positive linear relationship between *x* and *y* with a slope of 0.3’ (figure 1*b*).
And so on.

Figure 1*b* allows for a considerably larger number of permissible situations (which is obvious as it allows for any positive linear relationship). When formulating the hypothesis in figure 1*b*, we do not need to specify every single one of these permissible relationships. We can simply specify all possible positive slopes, which entails the set of permissible elements it includes.

**Figure 2.**
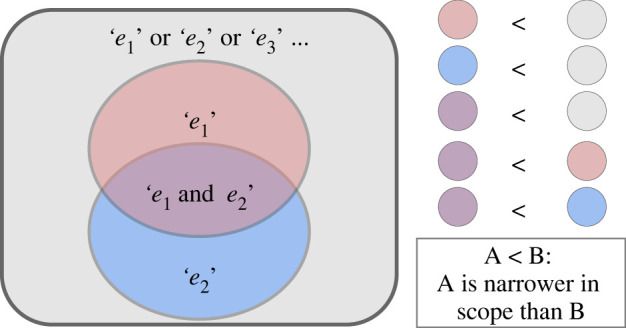
Scope as sets. *Left*: four different sets (grey, red, blue and purple) showing different elements which they contain. *Right*: a list of each colour explaining which set is a subset of the other (thereby being ‘narrower’).

That broader hypotheses have more elements in their sets entails some important properties. When we say **S** contains the elements *e*_1_, *e_2_*, and *e*_3_, the hypothesis is corroborated if *e*_1_ *or e*_2_ *or e*_3_ is the case. This means that the set requires only one of the elements to be corroborated for the hypothesis to be considered correct (i.e. the positive linear relationship needs to be 0.3 or 0.4 or 0.5). Contrastingly, we will later see cases when conjunctions of elements occur (i.e. both *e*_1_ *and* *e*_2_ are the case). When a conjunction occurs, in this formulation, the conjunction itself becomes an element in the set (i.e. ‘*e*_1_ and *e*_2_’ is a single element). Figure 2 illustrates how ‘*e*_1_ and *e*_2_’ is narrower than ‘*e*_1_’, and ‘*e*_1_’ is narrower than ‘*e*_1_ or *e*_2_’.^7^ This property relating to the conjunction being narrower than individual elements is explained in more detail in the pipeline selection section below.

### 4.2. Relationship selection

We move to A2, which is to show the different dimensions that a hypothesis scope can vary along. We have already seen an example of the first dimension of a hypothesis in figure 1, the *relationship selection*. Let **R** denote the set of all possible configurations of relationships that are permissible for the hypothesis to be considered true. For example, in the narrowest formulation above, there was one allowed relationship for the hypothesis to be true. Consequently, the size of **R** (denoted |**R**|) is one. As discussed above, in the second narrowest formulation (figure 1*b*), **R** has more possible relationships where it can still be considered true:

r_1_ = ‘a positive linear relationship of 0.1’
r_2_ = ‘a positive linear relationship of 0.2’
r_3_ = ‘a positive linear relationship of 0.3’.

And so on.

Additionally, even broader hypotheses will be compatible with more types of relationships. In figure 1*c*,*d*, nonlinear and negative relationships are also possible relationships included in **R**. For this broader statement to be affirmed, more elements are possible to be true. Thus if |**R**| is greater (i.e. contains more possible configurations for the hypothesis to be true), then the hypothesis is broader. Thus, the scope of relating to the relationship selection is specified by |**R**|. Finally, if **|R_H1_|** > **|R_H2_|**, then H_1_ is broader than H_2_ regarding the relationship selection.

Figure 1 is an example of the relationship narrowing. That the relationship became linear is only an example and does not necessitate a linear relationship or that this scope refers only to correlations. An alternative example of a relationship scope is a broad hypothesis where there is no knowledge about the distribution of some data. In such situations, one may assume a uniform relationship or a Cauchy distribution centred at zero. Over time the specific distribution can be hypothesized. Thereafter, the various parameters of the distribution can be hypothesized. At each step, the hypothesis of the distribution gets further specified to narrower formulations where a smaller set of possible relationships are included (see [47,48] for a more in-depth discussion about how specific priors relate to more narrow tests). Finally, while figure 1 was used to illustrate the point of increasingly narrow relationship hypotheses, it is more likely to expect the narrowest relationship, within fields such as psychology, to have considerable uncertainty and be formulated with confidence or credible intervals (i.e. we will rarely reach point estimates).

### 4.3. Variable selection

We have demonstrated that *relationship selection* can affect the scope of a hypothesis. Additionally, at least two other dimensions can affect the scope of a hypothesis: *variable selection* and *pipeline selection*. The variable selection in figure 1 was a single bivariate relationship (e.g. *x*'s relationship with *y*). However, it is not always the case that we know which variables will be involved. For example, in neuroimaging, we can be confident that one or more brain regions will be processing some information following a stimulus. Still, we might not be sure which brain region(s) this will be. Consequently, our hypothesis becomes broader because we have selected more variables. The relationship selection may be identical for each chosen variable, but the variable selection becomes broader. We can consider the following three hypotheses to be increasing in their scope:

H_1_: *x* relates to *y* with relationship **R**.
H_2_: *x*_1_ or *x_2_* relates to *y* with relationship **R**.
H_3_: *x*_1_ or *x*_2_ or *x*_3_ relates to *y* with relationship **R**.

For H_1_–H_3_ above, we assume that **R** is the same. Further, we assume that there is no interaction between these variables.

In the above examples, we have multiple *x* (*x*_1_, *x*_2_, *x*_3_, … , *x_n_*). Again, we can symbolize the variable selection as a non-empty set **XY**, containing either a single variable or many variables. Our motivation for designating it **XY** is that the variable selection can include multiple possibilities for both the independent variable (*x*) and the dependent variable (*y*). Like with relationship selection, we can quantify the broadness between two hypotheses with the size of the set **XY**. Consequently, |**XY**| denotes the total scope concerning variable selection. Thus, in the examples above |**XY**_H1_| < |**XY**_H2_| < |**XY**_H3_|. Like with relationship selection, hypotheses that vary in |**XY**| still meet the definition of a hypothesis.^8^

An obvious concern for many is that a broader **XY** is much easier to evaluate as correct. Generally, when |**XY_1_**| > |**XY_2_**|, there is a greater chance of spurious correlations when evaluating **XY_1_**. This concern is an issue relating to the evaluation of hypotheses (e.g. applying statistics to the evaluation), which will require additional assumptions relating to how to evaluate the hypotheses. Strategies to deal with this apply some correction or penalization for multiple statistical testing [49] or partial pooling and regularizing priors [50,51]. These strategies aim to evaluate a broader variable selection (*x*_1_ or *x*_2_) on equal or similar terms to a narrow variable selection (*x*_1_).

### 4.4. Pipeline selection

Scientific studies require decisions about how to perform the analysis. This scope considers transformations applied to the raw data (**XY_raw_**) to achieve some derivative (**XY**). These decisions can also involve selection procedures that drop observations deemed unreliable, standardizing, correcting confounding variables, or different philosophies. We can call the array of decisions and transformations used as the *pipeline*. A hypothesis varies in the number of pipelines:

H_1_: **XY** has a relationship(s) **R** with pipeline *p_1_*.
H_2_: **XY** has a relationship(s) **R** with pipeline *p_1_* or pipeline *p_2_*.
H_3_: **XY** has a relationship(s) **R** with pipeline *p_1_* or pipeline *p_2_*, or pipeline *p_3_*.

Importantly, the pipeline here considers decisions regarding how the hypothesis shapes the data collection and transformation. We do not consider this to include decisions made regarding the assumptions relating to the statistical inference as those relate to operationalizing the evaluation of the hypothesis and not part of the hypothesis being evaluated (these assumptions are like auxiliary hypotheses, which are assumed to be true but not explicitly evaluated).

Like with variable selection (**XY**) and relationship selection (**R**), we can see that pipelines impact the scope of hypotheses. Again, we can symbolize the pipeline selection with a set **P**. As previously, |**P**| will denote the dimension of the pipeline selection. In the case of pipeline selection, we are testing the same variables, looking for the same relationship, but processing the variables or relationships with different pipelines to evaluate the relationship. Consequently, |**P**_H1_| < |**P**_H2_| < |**P**_H3_|.

These issues regarding pipelines have received attention as the ‘garden of forking paths' [52]. Here, there are calls for researchers to ensure that their entire pipeline has been specified. Additionally, recent work has highlighted the diversity of results based on multiple analytical pipelines [53,54]. These results are often considered a concern, leading to calls that results should be pipeline resistant.

The wish for pipeline-resistant methods entails that hypotheses, in their narrowest form, are possible for all pipelines. Consequently, a narrower formulation will entail that this should not impact the hypothesis regardless of which pipeline is chosen. Thus the conjunction of pipelines is narrower than single pipelines. Consider the following three scenarios:

H_1_: **XY** has a relationship(s) **R** with pipeline *p_1_*.
H_2_: **XY** has a relationship(s) **R** with pipeline *p_1_* or pipeline *p_2_*.
H_3_: **XY** has a relationship(s) **R** with pipeline *p_1_ and* pipeline *p_2_*.

In this instance, since H_1_ is always true if H_3_ is true, thus H_3_ is a narrower formulation than H_1_. Consequently, |**P**_H3_| < |**P**_H1_| < |**P**_H2_|. Decreasing the scope of the pipeline dimension also entails the increase in conjunction of pipelines (i.e. creating pipeline-resistant methods) rather than just the reduction of disjunctional statements.

### 4.5. Combining the dimensions

In summary, we then have three different dimensions that independently affect the scope of the hypothesis. We have demonstrated the following general claim regarding hypotheses:

The variables **XY** have a relationship **R** with pipeline **P**.

And that the broadness and narrowness of a hypothesis depend on how large the three sets **XY**, **R** and **P** are. With this formulation, we can conclude that hypotheses have a scope that can be determined with a 3-tuple argument of (|**R**|, |**XY**|, |**P**|).

While hypotheses can be formulated along these three dimensions and generally aim to be reduced, it does not entail that these dimensions behave identically. For example, the relationship dimensions aim to reduce the number of elements as far as possible (e.g. to an interval). Contrastingly, for both variables and pipeline, the narrower hypothesis can reduce to single variables/pipelines or become narrower still and become conjunctions where all variables/pipelines need to corroborate the hypothesis (i.e. regardless of which method one follows, the hypothesis is correct).

## 5. Additional possible dimensions

No commitment is being made about the exhaustive nature of there only being three dimensions that specify the hypothesis scope. Other dimensions may exist that specify the scope of a hypothesis. For example, one might consider the pipeline dimension as two different dimensions. The first would consider the *experimental pipeline* dimension regarding all variables relating to the experimental setup to collect data, and the latter would be the *analytical pipeline* dimension regarding the data analysis of any given data snapshot. Another possible dimension is adding the number of situations or contexts under which the hypothesis is valid. For example, any restraint such as ‘in a vacuum’, ‘under the speed of light’, or ‘in healthy human adults' could be considered an additional dimension of the hypothesis. There is no objection to whether these should be additional dimensions of the hypothesis. However, as stated above, these usually follow from the boundary conditions of the theory.

## 6. Specifying the scope versus assumptions

We envision that this framework can help hypothesis-makers formulate hypotheses (in research plans, registered reports, preregistrations etc.). Further, using this framework while formulating hypotheses can help distinguish between auxiliary hypotheses and parts of the scientific hypothesis being tested. When writing preregistrations, it can frequently occur that some step in the method has two alternatives (e.g. a preprocessing step), and there is not yet reason to choose one over the other, and the researcher needs to make a decision. These following scenarios are possible:

1. *Narrow pipeline scope*. The researcher evaluates the hypothesis with both pipeline variables (i.e. H holds for both *p_1_* and *p_2_* where *p_1_* and *p_2_* can be substituted with each other in the pipeline).
2. *Broad pipeline scope.* The researcher evaluates the hypothesis with both pipeline variables, and only one needs to be correct (i.e. H holds for either *p_1_* or *p_2_* where *p_1_* and *p_2_* can be substituted with each other in the pipeline). The result of this experiment may help motivate choosing either *p_1_* or *p*_2_ in future studies.
3. *Auxiliary hypothesis.* Based on some reason (e.g. convention), the researcher assumes *p_1_* and evaluates *H* assuming *p_1_* is true.

Here we see that the same pipeline step can be part of either the auxiliary hypotheses or the pipeline scope. This distinction is important because if (3) is chosen, the decision becomes an *assumption* that is not explicitly tested by the hypothesis. Consequently, a researcher confident in the hypothesis may state that the auxiliary hypothesis *p_1_* was incorrect, and they should retest their hypothesis using different assumptions. In the cases where this decision is part of the pipeline scope, the hypothesis is intertwined with this decision, removing the eventual wiggle-room to reject auxiliary hypotheses that were assumed. Furthermore, starting with broader pipeline hypotheses that gradually narrow down can lead to a more well-motivated protocol for approaching the problem. Thus, this framework can help researchers while writing their hypotheses in, for example, preregistrations because they can consider when they are committing to a decision, assuming it, or when they should perhaps test a broader hypothesis with multiple possible options (discussed in more detail in §11 below).

## 7. The reduction of scope in hypothesis space

Having established that different scopes of a hypothesis are possible, we now consider how the hypotheses change over time. In this section, we consider how the scope of the hypothesis develops *ideally* within science.

**Figure 3.**
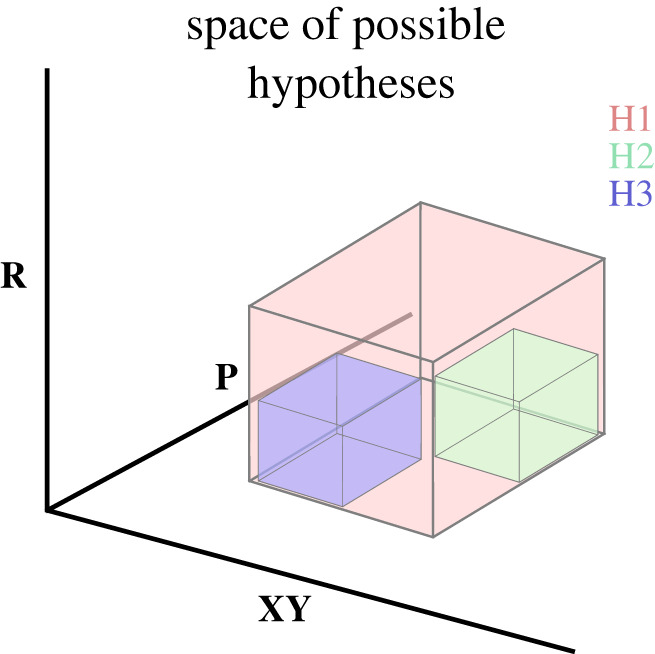
Example of hypothesis space. The hypothesis scope is expressed as cuboids in three dimensions (relationship (**R**), variable (**XY**), pipeline (**P**)). The hypothesis space is the entire possible space within the three dimensions. Three hypotheses are shown in the hypothesis space (H_1_, H_2_, H_3_). H_2_ and H_3_ are subsets of H_1_.

Consider a new research question. A large number of hypotheses are possible. Let us call this set of all possible hypotheses the *hypothesis space*. Hypotheses formulated within this space can be narrower or broader based on the dimensions discussed previously (figure 3).

After the evaluation of the hypothesis with the scientific process, the hypothesis will be accepted or rejected.^9^ The evaluation could be done through falsification or via verification, depending on the philosophy of science commitments. Thereafter, other narrower formulations of the hypothesis can be formulated by reducing the relationship, variable or pipeline scope. If a narrower hypothesis is accepted, more specific details about the subject matter are known, or a theory has been refined in greater detail. A narrower hypothesis will entail a more specific relationship, variable or pipeline detailed in the hypothesis. Consequently, hypotheses linked to each other in this way will become narrower over time along one or more dimensions. Importantly, considering that the conjunction of elements is narrower than single elements for pipelines and variables, this process of narrower hypotheses will lead to more general hypotheses (i.e. they have to be applied in all conditions and yield less flexibility when they do not apply).^10^

Considering that the scopes of hypotheses were defined as sets above, some properties can be deduced from this framework about how narrower hypotheses relate to broader hypotheses. Let us consider three hypotheses (H_1_, H_2_, and H_3_; figure 3). H_2_ and H_3_ are non-overlapping subsets of H_1_. Thus H_2_ and H_3_ are both narrower in scope than H_1_. Thus the following is correct:

P1: If H_1_ is false, then H_2_ is false, and H_2_ does not need to be evaluated.
P2: If H_2_ is true, then the broader H_1_ is true, and H_1_ does not need to be evaluated.
P3: If H_1_ is true and H_2_ is false, some other hypothesis H_3_ of similar scope to H_2_ is possible.

For example, suppose H_1_ is ‘there is a relationship between *x* and *y*’, H_2_ is ‘there is a positive relationship between *x* and *y*’, and H_3_ is ‘a negative relationship between *x* and *y*’. In that case, it becomes apparent how each of these follows.^11^ Logically, many deductions from set theory are possible but will not be explored here. Instead, we will discuss two additional consequences of hypothesis scopes: scientific novelty and applications for the researcher who formulates a hypothesis.

P1–P3 have been formulated as hypotheses being true or false. In practice, hypotheses are likely evaluated probabilistically (e.g. ‘H_1_ is likely’ or ‘there is evidence in support of H_1_’). In these cases, P1–P3 can be rephrased to account for this by substituting true/false with statements relating to evidence. For example, P2 could read: ‘If there is evidence in support of H_2_, then there is evidence in support of H_1_, and H_1_ does not need to be evaluated’.

## 8. Scientific novelty as the reduction of scope

Novelty is a key concept that repeatedly occurs in multiple aspects of the scientific enterprise, from funding to publishing [55]. Generally, scientific progress establishes novel results based on some new hypothesis. Consequently, the new hypothesis for the novel results must be narrower than previously established knowledge (i.e. the size of the scopes is reduced). Otherwise, the result is trivial and already known (see P2 above). Thus, scientific work is novel if the scientific process produces a result based on hypotheses with either a smaller |**R**|, |**XY**|, or |**P**| compared to previous work.

This framework of dimensions of the scope of a hypothesis helps to demarcate when a hypothesis and the subsequent result are novel. If previous studies have established evidence for **R_1_** (e.g. there is a positive relationship between *x* and *y*), a hypothesis will be novel if and only if it is narrower than **R_1_**. Thus, if **R_2_** is narrower in scope than **R_1_** (i.e. |**R_2_**| < |**R_1_**|), **R_2_** is a novel hypothesis.

Consider the following example. Study 1 hypothesizes, ‘There is a positive relationship between *x* and *y*’. It identifies a linear relationship of 0.6. Next, Study 2 hypothesizes, ‘There is a specific linear relationship between *x* and *y* that is 0.6’. Study 2 also identifies the relationship of 0.6. Since this was a narrower hypothesis, Study 2 is novel despite the same result. Frequently, researchers claim that they are the first to demonstrate a relationship. Being the first to demonstrate a relationship is not the final measure of novelty. Having a narrower hypothesis than previous researchers is a sign of novelty as it further reduces the hypothesis space.

Finally, it should be noted that novelty is not the only objective of scientific work. Other attributes, such as improving the certainty of a current hypothesis (e.g. through replications), should not be overlooked. Additional scientific explanations and improved theories are other aspects. Additionally, this definition of novelty relating to hypothesis scope does not exclude other types of novelty (e.g. new theories or paradigms).

## 9. How broad should a hypothesis be?

Given the previous section, it is elusive to conclude that the hypothesis should be as narrow as possible as it entails maximal knowledge gain and scientific novelty when formulating hypotheses. Indeed, many who advocate for daring or risky tests seem to hold this opinion. For example, Meehl [46] argues that we should evaluate theories based on point (or interval) prediction, which would be compatible with very narrow versions of relationships. We do not necessarily think that this is the most fruitful approach. In this section, we argue that hypotheses should aim to be *narrower than current knowledge*, but *too narrow may be problematic*.

Let us consider the idea of confirmatory analyses. These studies will frequently keep the previous hypothesis scopes regarding **P** and **XY** but aim to become more specific regarding **R** (i.e. using the same method and the same variables to detect a more specific relationship). A very daring or narrow hypothesis is to minimize **R** to include the fewest possible relationships. However, it becomes apparent that simply pursuing specificness or daringness is insufficient for selecting relevant hypotheses. Consider a hypothetical scenario where a researcher believes virtual reality use leads people to overestimate the amount of exercise they have done. If unaware of previous studies on this project, an apt hypothesis is perhaps ‘increased virtual reality usage correlates with a less accuracy of reported exercise performed’ (i.e. **R** is broad). However, a more specific and more daring hypothesis would be to specify the relationship further. Thus, despite not knowing if there is a relationship at all, a more daring hypothesis could be: ‘for every 1 h of virtual reality usage, there will be, on average, a 0.5% decrease in the accuracy of reported exercise performed’ (i.e. **R** is narrow). We believe it would be better to establish the broader hypothesis in any scenario here for the first experiment. Otherwise, if we fail to confirm the more specific formulation, we could reformulate another equally narrow relative to the broader hypothesis. This process of tweaking a daring hypothesis could be pursued *ad infinitum*. Such a situation will neither quickly identify the true hypothesis nor effectively use limited research resources.

By first discounting a broader hypothesis that there is no relationship, it will automatically discard all more specific formulations of that relationship in the hypothesis space. Returning to figure 3, it will be better to establish H_1_ before attempting H_2_ or H_3_ to ensure the correct area in the hypothesis space is being investigated. To provide an analogy: when looking for a needle among hay, first identify which farm it is at, then which barn, then which haystack, then which part of the haystack it is at before we start picking up individual pieces of hay. Thus, it is preferable for both pragmatic and cost-of-resource reasons to formulate sufficiently broad hypotheses to navigate the hypothesis space effectively.

Conversely, formulating too broad a relationship scope in a hypothesis when we already have evidence for narrower scope would be superfluous research (unless the evidence has been called into question by, for example, not being replicated). If multiple studies have supported the hypothesis ‘there is a 20-fold decrease in mortality after taking some medication M’, it would be unnecessary to ask, ‘Does M have any effect?’.

Our conclusion is that the appropriate scope of a hypothesis, and its three dimensions, follow a Goldilocks-like principle where too broad is superfluous and not novel, while too narrow is unnecessary or wasteful. Considering the scope of one's hypothesis and how it relates to previous hypotheses' scopes ensures one is asking appropriate questions.

Finally, there has been a recent trend in psychology that hypotheses should be formal [38,56–60]. Formal theories are precise since they are mathematical formulations entailing that their interpretations are clear (non-ambiguous) compared to linguistic theories. However, this literature on formal theories often refers to ‘precise predictions’ and ‘risky testing’ while frequently referencing Meehl, who advocates for narrow hypotheses (e.g. [38,56,59]). While perhaps not intended by any of the proponents, one interpretation of some of these positions is that hypotheses derived from formal theories will be narrow hypotheses (i.e. the quality of being ‘precise’ can mean narrow hypotheses with risky tests and non-ambiguous interpretations simultaneously). However, the benefit from the clarity (non-ambiguity) that formal theories/hypotheses bring also applies to broad formal hypotheses as well. They can include explicit but formalized versions of uncertain relationships, multiple possible pipelines, and large sets of variables. For example, a broad formal hypothesis can contain a hyperparameter that controls which distribution the data fit (broad relationship scope), or a variable could represent a set of formalized explicit pipelines (broad pipeline scope) that will be tested. In each of these instances, it is possible to formalize non-ambiguous broad hypotheses from broad formal theories that do not yet have any justification for being overly narrow. In sum, our argumentation here stating that hypotheses should not be too narrow is not an argument against formal theories but rather that hypotheses (derived from formal theories) do not necessarily have to be narrow.

## 10. Other approaches to categorizing hypotheses

The framework we present here is a way of categorizing hypotheses into (at least) three dimensions regarding the hypothesis scope, which we believe is accessible to researchers and help link scientific work over time while also trying to remain neutral with regard to a specific philosophy of science. Our proposal does not aim to be antagonistic or necessarily contradict other categorizing schemes—but we believe that our framework provides benefits.

One recent categorization scheme is the Theoretical (T), Auxiliary (A), Statistical (S) and Inferential (I) assumption model (together becoming the TASI model) [61,62]. Briefly, this model considers theory to generate theoretical hypotheses. To translate from theoretical unobservable terms (e.g. personality, anxiety, mass), auxiliary assumptions are needed to generate an empirical hypothesis. Statistical assumptions are often needed to test the empirical hypothesis (e.g. what is the distribution, is it skewed or not) [61,62]. Finally, additional inferential assumptions are needed to generalize to a larger population (e.g. was there a random and independent sampling from defined populations). The TASI model is insightful and helpful in highlighting the distance between a theory and the observation that would corroborate/contradict it. Part of its utility is to bring auxiliary hypotheses into the foreground, to improve comparisons between studies and improve theory-based interventions [63,64].

We do agree with the importance of being aware of or stating the auxiliary hypotheses, but there are some differences between the frameworks. First, the number of auxiliary assumptions in TASI can be several hundred [62], whereas our framework will consider some of them as part of the pipeline dimension. Consider the following four assumptions: ‘the inter-stimulus interval is between 2000 ms and 3000 ms', ‘the data will be z-transformed’, ‘subjects will perform correctly’, and ‘the measurements were valid’. According to the TASI model, all these will be classified similarly as auxiliary assumptions. Contrarily, within our framework, it is possible to consider the first two as part of the pipeline dimension and the latter two as auxiliary assumptions, and consequently, the first two become integrated as part of the hypothesis being tested and the latter two auxiliary assumptions. A second difference between the frameworks relates to non-theoretical studies (convenience, applied or atheoretical). Our framework allows for the possibility that the hypothesis space generated by theoretical and convenience studies can interact and inform each other *within the same framework*. Contrarily, in TASI, the theory assumptions no longer apply, and a different type of hypothesis model is needed; these assumptions must be replaced by another group of assumptions (where ‘substantive application assumptions' replace the T and the A, becoming SSI) [61]. Finally, part of our rationale for our framework is to be able to link and track hypotheses and hypothesis development together over time, so our classification scheme has different utility.

Another approach which has some similar utility to this framework is theory construction methodology (TCM) [57]. The similarity here is that TCM aims to be a practical guide to improve theory-making in psychology. It is an iterative process which relates theory, phenomena and data. Here hypotheses are not an explicit part of the model. However, what is designated as ‘proto theory’ could be considered a hypothesis in our framework as they are a product of abduction, shaping the theory space. Alternatively, what is deduced to evaluate the theory can also be considered a hypothesis. We consider both possible and that our framework can integrate with these two steps, especially since TCM does not have clear guidelines for how to do each step.

## 11. From theory to practice: implementing this framework

We believe that many practising researchers can relate to many aspects of this framework. But, how can a researcher translate the above theoretical framework to their work? The utility of this framework lies in bringing these three scopes of a hypothesis together and explaining how each can be reduced. We believe researchers can use this framework to describe their current practices more clearly. Here we discuss how it can be helpful for researchers when formulating, planning, preregistering, and discussing the evaluation of their scientific hypotheses. These practical implications are brief, and future work can expand on the connection between the full interaction between hypothesis space and scope. Furthermore, both authors have the most experience in cognitive neuroscience, and some of the practical implications may revolve around this type of research and may not apply equally to other fields.

### 11.1. Helping to form hypotheses

Abduction, according to Peirce, is a hypothesis-making exercise [22,26–28]. Given some observations, a general testable explanation of the phenomena is formed. However, when making the hypothesis, this statement will have a scope (either explicitly or implicitly). Using our framework, the scope can become explicit. The hypothesis-maker can start with ‘The variables **XY** have a relationship **R** with pipeline **P**’ as a scaffold to form the hypothesis. From here, the hypothesis-maker can ‘fill in the blanks’, explicitly adding each of the scopes. Thus, when making a hypothesis via abduction and using our framework, the hypothesis will have an explicit scope when it is made. By doing this, there is less chance that a formulated hypothesis is unclear, ambiguous, and needs amending at a later stage.

### 11.2. Assisting to clearly state hypotheses

A hypothesis is not just formulated but also communicated. Hypotheses are stated in funding applications, preregistrations, registered reports, and academic articles. Further, preregistered hypotheses are often omitted or changed in the final article [11], and hypotheses are not always explicitly stated in articles [12]. How can this framework help to make better hypotheses? Similar to the previous point, filling in the details of ‘The variables **XY** have a relationship **R** with pipeline **P**’ is an explicit way to communicate the hypothesis. Thinking about each of these dimensions should entail an appropriate explicit scope and, hopefully, less variation between preregistered and reported hypotheses. The hypothesis does not need to be a single sentence, and details of **XY** and **P** will often be developed in the methods section of the text. However, using this template as a starting point can help ensure the hypothesis is stated, and the scope of all three dimensions has been communicated.

### 11.3. Helping to promote explicit and broad hypotheses instead of vague hypotheses

There is an important distinction between vague hypotheses and broad hypotheses, and this framework can help demarcate between them. A vague statement would be: ‘We will quantify depression in patients after treatment’. Here there is uncertainty relating to how the researcher will go about doing the experiment (i.e. how will depression be quantified?). However, a broad statement can be uncertain, but the uncertainty is part of the hypothesis: ‘Two different mood scales (S_1_ or S_2_) will be given to patients and test if only one (or both) changed after treatment’. This latter statement is transparently saying ‘S_1_ or S_2_’ is part of a broad hypothesis—the uncertainty is whether the two different scales are quantifying the same construct. We keep this uncertainty within the broad hypothesis, which will get evaluated, whereas a vague hypothesis has uncertainty as part of the interpretation of the hypothesis. This framework can be used when formulating hypotheses to help be broad (where needed) but not vague.

### 11.4. Which hypothesis should be chosen?

When considering the appropriate scope above, we argued for a Goldilocks-like principle of determining the hypothesis that is not too broad or too narrow. However, when writing, for example, a preregistration, how does one identify this sweet spot? There is no easy or definite universal answer to this question. However, one possible way is first to identify the **XY**, **R**, and **P** of previous hypotheses. From here, identify what a non-trivial step is to improve our knowledge of the research area. So, for example, could you be more specific about the exact nature of the relationship between the variables? Does the pipeline correspond to today's scientific standards, or were some suboptimal decisions made? Is there another population that you think the previous result also applies to? Do you think that maybe a more specific construct or subpopulation might explain the previous result? Could slightly different constructs (perhaps easier to quantify) be used to obtain a similar relationship? Are there even more constructs to which this relationship should apply simultaneously? Are you certain of the direction of the relationship? Answering affirmatively to any of these questions will likely make a hypothesis narrower and connect to previous research while being clear and explicit. Moreover, depending on the research question, answering any of these may be sufficiently narrow to be a non-trivial innovation. However, there are many other ways to make a hypothesis narrower than these guiding questions.

### 11.5. The confirmatory–exploratory continuum

Research is often dichotomized into confirmatory (testing a hypothesis) or exploratory (without *a* *priori* hypotheses). With this framework, researchers can consider how their research acts on some hypothesis space. Confirmatory and exploratory work has been defined in terms of how each interacts with the researcher's degrees of freedom (where confirmatory aims to reduce while exploratory utilizes them [30]). Both broad confirmatory and narrow exploratory research are possible using this definition and possible within this framework. How research interacts with the hypothesis space helps demarcate it. For example, if a hypothesis reduces the scope, it becomes more confirmatory, and trying to understand data given the current scope would be more exploratory work. This further could help demarcate when exploration is useful. Future theoretical work can detail how different types of research impact the hypothesis space in more detail.

### 11.6. Understanding when multiverse analyses are needed

Researchers writing a preregistration may face many degrees of freedom they have to choose from, and different researchers may motivate different choices. If, when writing such a preregistration, there appears to be little evidential support for certain degrees of freedom over others, the researcher is left with the option to either make more auxiliary assumptions or identify when an investigation into the pipeline scope is necessary by conducting a multiverse analysis that tests the impact of the different degrees of freedom on the result (see [8]). Thus, when applying this framework to explicitly state what pipeline variables are part of the hypothesis or an auxiliary assumption, the researcher can identify when it might be appropriate to conduct a multiverse analysis because they are having difficulty formulating hypotheses.

### 11.7. Describing novelty

Academic journals and research funders often ask for novelty, but the term ‘novelty’ can be vague and open to various interpretations [55]. This framework can be used to help justify the novelty of research. For example, consider a scenario where a previous study has established a psychological construct (e.g. well-being) that correlates with a certain outcome measure (e.g. long-term positive health outcomes). This framework can be used to explicitly justify novelty by (i) providing a more precise understanding of the relationship (e.g. linear or linear–plateau) or (ii) identifying more specific variables related to well-being or health outcomes. Stating how some research is novel is clearer than merely stating that the work is novel. This practice might even help journals and funders identify what type of novelty they would like to reward. In sum, this framework can help identify and articulate how research is novel.

### 11.8. Help to identify when standardization of pipelines is beneficial or problematic to a field

Many consider standardization in a field to be important for ensuring the comparability of results. Standardization of methods and tools entails that the pipeline **P** is identical (or at least very similar) across studies. However, in such cases, the standardized pipeline becomes an auxiliary assumption representing all possible pipelines. Therefore, while standardized pipelines have their benefits, this assumption becomes broader without validating (e.g. via multiverse analysis) which pipelines a standardized **P** represents. In summary, because this framework helps distinguish between auxiliary assumptions and explicit parts of the hypothesis and identifies when a multiverse analysis is needed, it can help determine when standardizations of pipelines are representative (narrower hypotheses) or assumptive (broader hypotheses).

## 12. Conclusion

Here, we have argued that the scope of a hypothesis is made up of three dimensions: the relationship (**R**), variable (**XY**) and pipeline (**P**) selection. Along each of these dimensions, the scope can vary. Different types of scientific enterprises will often have hypotheses that vary the size of the scopes. We have argued that this focus on the scope of the hypothesis along these dimensions helps the hypothesis-maker formulate their hypotheses for preregistrations while also helping demarcate auxiliary hypotheses (assumed to be true) from the hypothesis (those being evaluated during the scientific process).

Hypotheses are an essential part of the scientific process. Considering what type of hypothesis is sufficient or relevant is an essential job of the researcher that we think has been overlooked. We hope this work promotes an understanding of what a hypothesis is and how its formulation and reduction in scope is an integral part of scientific progress. We hope it also helps clarify how broad hypotheses need not be vague or inappropriate.

Finally, we applied this idea of scopes to scientific progress and considered how to formulate an appropriate hypothesis. We have also listed several ways researchers can practically implement this framework today. However, there are other practicalities of this framework that future work should explore. For example, it could be used to differentiate and demarcate different scientific contributions (e.g. confirmatory studies, exploration studies, validation studies) with how their hypotheses interact with the different dimensions of the hypothesis space. Further, linking hypotheses over time within this framework can be a foundation for open hypothesis-making by promoting explicit links to previous work and detailing the reduction of the hypothesis space. This framework helps quantify the contribution to the hypothesis space of different studies and helps clarify what aspects of hypotheses can be relevant at different times.

## Data Availability

This article has no additional data.

## Footnotes

1 While this is our intention, we cannot claim that every theory has been accommodated.

2 Similar requirements of science being able to evaluate the hypothesis can be found in pragmatism [22], logical positivism [23] and falsification [24].

3 Although when making inferences about a failed evaluation of a scientific hypothesis it is possible, due to underdetermination, to reject the auxiliary hypothesis instead of rejecting the hypothesis. However, that rejection occurs at a later inference stage. The evaluation using the scientific method aims to test the scientific hypothesis, not the auxiliary assumptions.

4 Although some have argued that this practice is not as problematic or questionable (see [34,35]).

5 Alternatively, theories sometimes expand their boundary conditions. A theory that was previously about ‘humans' can be used with a more inclusive boundary condition. Thus it is possible for the hypothesis-maker to use a theory about humans (decision making) and expand it to fruit flies or plants (see [43]).

6 A similarity exists here with Popper, where he uses set theory in a similar way to compare theories (not hypotheses). Popper also discusses how theories with overlapping sets but neither is a subset are also comparable (see [24, §§32–34]). We do not exclude this possibility but can require additional assumptions.

7 When this could be unclear, we place the element within quotation marks.

8 Here, we have assumed that there is no interaction between these variables in variable selection. If an interaction between *x*_1_ and *x*_2_ is hypothesized, this should be viewed as a different variable compared to ‘*x*_1_ or *x*_2_’. The motivation behind this is because the hypothesis ‘*x*_1_ or *x*_2_’ is not a superset of the interaction (i.e. ‘*x*_1_ or *x*_2_’ is not necessarily true when the interaction is true). The interaction should, in this case, be considered a third variable (e.g. I(*x*_1_, *x*_2_)) and the hypothesis ‘*x*_1_ or *x*_2_ or I(*x*_1_, *x*_2_)’ is broader than ‘*x*_1_ or *x*_2_’.

9 Or possibly ambiguous or inconclusive.

10 This formulation of scope is compatible with different frameworks from the philosophy of science. For example, by narrowing the scope would in a Popperian terminology mean prohibiting more *basic statements* (thus a narrower hypothesis has a higher degree of falsifiability). The reduction of scope in the relational dimension would in Popperian terminology mean increase in *precision* (e.g. a circle is more precise than an ellipse since circles are a subset of possible ellipses), whereas reduction in variable selection and pipeline dimension would mean increase *universality* (e.g. ‘all heavenly bodies' is more universal than just ‘planets') [24]. For Meehl the reduction of the relationship dimension would amount to decreasing the *relative tolerance of a theory* to the *Spielraum* [46]*.*

11 If there is no relationship between *x* and *y*, we do not need to test if there is a positive relationship. If we know there is a positive relationship between *x* and *y*, we do not need to test if there is a relationship. If we know there is a relationship but there is not a positive relationship, then it is possible that they have a negative relationship.
